# Immunohistochemical Characterization of 14-3-3σ Expression in Canine Primary Pulmonary Carcinomas: A Preliminary Study

**DOI:** 10.3390/vetsci13090926

**Published:** 2026-09-09

**Authors:** Alexandra Morar, Corina Toma, Diana Bochynska, Dragos Hodor, Andrada Negoescu, Cristina Diana Borfalau, Stefana Prica, Claudiu Gal, Cornel Catoi

**Affiliations:** 1 Department of Veterinary Pathology, Faculty of Veterinary Medicine, University of Agricultural Sciences and Veterinary Medicine Cluj-Napoca, 400372 Cluj-Napoca, Romania; alexandra.morar@usamvcluj.ro (A.M.); corina.toma@usamvcluj.ro (C.T.); dragos.hodor@usamvcluj.ro (D.H.); andrada.negoescu@usamvcluj.ro (A.N.); cristina-diana.borfalau@usamvcluj.ro (C.D.B.); stefana.prica@usamvcluj.ro (S.P.); cornel.catoi@usamvcluj.ro (C.C.); 2 Department of Veterinary Pathology, Ross University School of Veterinary Medicine, Basseterre 00265, Saint Kitts and Nevis; 3 Department of Veterinary Pathology, Synevovet, Industriilor 25, Ilfov, 077040 Chiajna, Romania; gal.claudiu@gmail.com

**Keywords:** CPPC, immunohistochemistry, 14-3-3σ, Ki-67, cell-cycle regulation, veterinary oncology

## Abstract

14-3-3σ is a cell-cycle regulatory protein implicated in tumor development and progression, but its expression in canine primary pulmonary carcinomas (CPPC) has not been characterized. This study investigated 14-3-3σ immunohistochemical expression in primary pulmonary carcinomas and its relationship with histological grade, proliferative activity, mitotic count, and tumor volume. Ten CPPCs were examined and graded according to established criteria. Immunohistochemical expression of 14-3-3σ was evaluated using a semiquantitative scoring system, while Ki-67 labeling was assessed by calculating the labeling index. Nine of 10 tumors (90%) showed 14-3-3σ immunoreactivity, which was predominantly multifocal, heterogeneous, and cytoplasmic; nuclear immunoreactivity was observed, particularly in areas showing strong cytoplasmic staining. Exploratory correlation analyses did not identify statistically significant associations between the 14-3-3σ immunohistochemical score and histological grade, Ki-67 labeling index, mitotic count, or estimated tumor volume. The Ki-67 labeling index ranged from 20.4% to 89.5% (median, 35.6%). These findings provide preliminary descriptive data on 14-3-3σ immunoreactivity in CPPCs. Larger studies incorporating quantitative assessment and clinical follow-up are warranted to determine its clinicopathological and prognostic significance.

## Simple Summary

Canine primary pulmonary carcinoma (CPPC) is a rare type of lung cancer in dogs, and understanding the molecular mechanisms behind it is important for better diagnosis and treatment. This preliminary study investigated the presence of 14-3-3σ, a specific protein known to regulate cell division and play a role in the progression of various human cancers. Researchers analyzed tumor samples from 10 dogs with CPPC to determine if this protein was present and to see if its expression levels were related to the tumor’s grade, size, or how fast the tumor cells were multiplying (using a proliferation marker called Ki-67). The results showed that the 14-3-3σ protein was present in 90% (9 out of 10) of the examined lung tumors, with the protein mostly found in the cytoplasm of the cells in an uneven, scattered pattern. However, there was no statistically significant relationship between the amount of 14-3-3σ protein and the tumor’s size, grade, or cellular proliferation rate. Ultimately, this study provides the first evidence that canine lung tumors express the 14-3-3σ protein, though larger future studies are needed to determine if it can be used to predict how aggressive the cancer will be.

## 1. Introduction

Canine primary pulmonary carcinoma (CPPC) is an uncommon epithelial neoplasm that shares several histopathologic and molecular characteristics with human non-small-cell lung carcinoma [1,2,3,4,5]. Although histopathologic evaluation remains essential for diagnosis and classification, tumors with similar morphologic features may display variable biological behavior, highlighting the need for additional biomarkers to refine tumor characterization.

Several immunohistochemical and molecular biomarkers have been investigated in CPPCs to improve understanding of their biological behavior. These include markers of cellular proliferation, such as Ki-67 and proliferating cell nuclear antigen (PCNA), tumor suppressor proteins including p53, growth factor signaling molecules such as epidermal growth factor receptor (EGFR), inflammatory mediators including cyclooxygenase-2 (COX-2), and proteins involved in cell adhesion and epithelial differentiation [6,7,8]. While these studies have expanded the molecular characterization of CPPCs, proteins involved in the DNA damage response and cell-cycle checkpoint regulation remain largely unexplored.

Cell-cycle dysregulation is a fundamental hallmark of cancer, contributing to uncontrolled proliferation, genomic instability, and tumor progression. Among the proteins coordinating these processes, the 14-3-3 family comprises a group of highly conserved regulatory molecules involved in cell-cycle progression, apoptosis, signal transduction, DNA damage response, and cellular differentiation [9,10]. Of the seven mammalian isoforms, 14-3-3σ (stratifin; SFN) is unique because its expression is largely restricted to epithelial tissues and is transcriptionally regulated by the p53 pathway [10]. Following DNA damage, 14-3-3σ promotes G2/M cell-cycle arrest by sequestering cyclin B1–CDC2 complexes within the cytoplasm, thereby preventing premature entry into mitosis and maintaining genomic stability [11].

Altered expression of 14-3-3σ has been reported in numerous human epithelial malignancies, including breast, lung, bladder, gastric, colorectal, and head and neck carcinomas [9,10]. Depending on the tumor type, reduced expression has been associated with promoter hypermethylation and loss of tumor suppressor function, whereas overexpression has been linked to tumor progression, invasion, metastasis, and poor clinical outcome [10]. In human non-small-cell lung carcinoma, altered 14-3-3σ expression has been reported in association with histologic subtype and clinicopathologic features, suggesting a potential role in pulmonary carcinogenesis [12,13].

In veterinary medicine, studies evaluating 14-3-3σ remain limited. Immunohistochemical expression has been characterized in normal canine tissues, confirming its predominant epithelial localization, and altered expression has been described in canine mammary tumors [14], as well as renal and gastric carcinomas [15,16]. However, whether 14-3-3σ is expressed in CPPCs and whether its expression is associated with tumor proliferative activity remains undetermined.

Assessment of 14-3-3σ expression in conjunction with the Ki-67 labeling index may provide further insight into the relationship between cell-cycle checkpoint regulation and tumor proliferative activity. Ki-67 is a well-established marker of cellular proliferation that has been widely applied in both human and veterinary oncology to estimate tumor growth fraction and biological aggressiveness [17,18].

To the best of our knowledge, the immunohistochemical expression and potential biological significance of 14-3-3σ have not previously been investigated in CPPC. Therefore, the aim of this preliminary study was to characterize the immunohistochemical expression of 14-3-3σ in CPPC, evaluate its association with tumor proliferative activity as assessed by the Ki-67 labeling index, and explore potential associations with selected clinicopathologic parameters.

## 2. Materials and Methods

### 2.1. Case Selection

Cases of CPPC were retrospectively retrieved from the archives of a private diagnostic laboratory. Twelve (*n* = 12) cases were initially identified as potentially eligible based on the available histopathological and clinical records. All tumor specimens included in this study were obtained from surgical pulmonary lobectomy specimens. The inclusion criteria were as follows: (1) a diagnosis of primary pulmonary carcinoma supported by histopathologic characteristics, thyroid transcription factor-1 (TTF-1) immunoreactivity, and the available submitted clinical history; (2) availability of adequate formalin-fixed, paraffin-embedded (FFPE) tumor tissue for subsequent immunohistochemical evaluation; and (3) availability of clinical information, including patient age, sex, breed, and tumor dimensions, recorded during gross examination (trimming) of the formalin-fixed specimens. Cases were excluded when adequate tumor tissue was unavailable for analysis, when immunohistochemical staining was considered technically non-evaluable because the corresponding positive control failed, or when metastatic disease or another primary tumor was reported in the submitted clinical history.

### 2.2. Histological Evaluation

Hematoxylin and eosin (H&E)-stained sections from each case were reviewed to confirm the diagnosis and evaluate tumor characteristics. Tumors were histologically classified according to the criteria described for respiratory tract tumors in domestic animals [19]. CPPCs were graded according to a previously described histological scoring system, incorporating overall differentiation, nuclear pleomorphism, mitotic count, nucleolar size, tumor necrosis, tumor fibrosis, and tumor demarcation. The individual scores were summed to obtain a total histological score, with tumors classified as Grade I (≤8.5), Grade II (9.0–14.0), or Grade III (≥14.5) [20]. Mitotic activity was assessed digitally using QuPath 0.7.0 on whole-slide images on a total analyzed area of 2.37 mm^2^, which was considered to correspond to the 10 high-power fields (HPF) specified in the original grading system. Vascular invasion was recorded when neoplastic cells were identified within vascular lumina on H&E-stained sections; blood and lymphatic vessels were not systematically differentiated in the present study.

### 2.3. Immunohistochemistry

Immunohistochemical evaluation was performed on formalin-fixed, paraffin-embedded (FFPE) canine pulmonary tissue sections for the detection of MCK, TTF-1, 14-3-3σ, and Ki-67. Depending on the antibody, staining was performed using either an automated platform or a manual protocol. Information on the primary antibodies, including manufacturer, clone, dilution, and antigen-retrieval conditions, is provided in Table 1.

MCK and TTF-1 were detected using an automated Leica BOND-MAX system (Leica Biosystems, Melbourne, Australia; BOND-MAX series M2 12154). The antibodies used were anti-MCK, clone AE1/AE3 (catalogue no. PA0909-U), and anti-TTF-1, clone SPT24 (catalogue no. PA0364). Antigen retrieval and staining procedures were performed according to the manufacturer’s recommended conditions, using the BOND Polymer Refine Detection system (Leica Biosystems, catalogue no. NC0318637). Bronchiolar epithelial cells and type II pneumocytes served as internal positive controls for MCK and TTF-1, respectively. MCK immunoreactivity was assessed based on cytoplasmic staining, whereas TTF-1 immunoreactivity was identified by nuclear staining of neoplastic cells. For both markers, staining was evaluated qualitatively according to the presence or absence of immunoreactivity.

Manual immunohistochemical staining was performed for 14-3-3σ and Ki-67. Sections were deparaffinized, rehydrated through graded alcohols, and subjected to heat-induced epitope retrieval using citrate buffer (pH 6.0) for 20 min. Endogenous peroxidase activity and non-specific protein binding were blocked using the corresponding reagents from the EnVision FLEX system (Agilent/Dako), according to the manufacturer’s instructions. Sections were then incubated overnight at 4 °C with the primary antibodies against 14-3-3σ, clone 5D7 (catalogue no. sc-100638), and Ki-67, clone MIB-1 (catalogue no. GA62661-2). Immunoreactivity was visualized using the EnVision FLEX detection system (Agilent Dako, Santa Clara, CA, USA), with 3,3′-diaminobenzidine (DAB) as the chromogen, followed by counterstaining with Mayer’s hematoxylin.

Basal cells of the airway epithelium served as internal positive controls for 14-3-3σ. For Ki-67, canine lymph node tissue was used as an external positive control, while lymphoid cells within bronchus-associated lymphoid tissue (BALT) provided an internal positive control. The expected staining pattern for 14-3-3σ was predominantly cytoplasmic, whereas Ki-67 immunoreactivity was characterized by nuclear staining. The 14-3-3σ antibody (clone 5D7) has previously been used for immunohistochemical detection of 14-3-3σ in normal and neoplastic mammary tissue and canine renal cell carcinomas [14,15].

Negative controls were included for all immunohistochemical procedures. For these controls, the primary antibody was omitted, while all subsequent incubation, detection, and visualization steps were performed under otherwise identical conditions.

The immunohistochemical expression of 14-3-3σ was evaluated according to a previously established semiquantitative method [15]. Two parameters were considered for each case: the proportion of neoplastic cells showing immunoreactivity and the intensity of staining. Two pathologists independently evaluated the entire tumor section while blinded to the histological grade, Ki-67 labeling index, and other clinicopathological data. The scores assigned independently by the two pathologists were subsequently compared, and discrepancies were resolved by consensus to establish the final score for each case. The proportion of positive cells was categorized into four groups: ≤10% (score 1; including cases with 0% positive cells), 11–50% (score 2), 51–80% (score 3), and >80% (score 4). Staining intensity was graded from 0 to 3, corresponding to negative, weak, moderate, and strong staining, respectively. An overall immunohistochemical score was subsequently obtained for each case by multiplying the scores assigned to the two parameters, resulting in a possible range of 0–12. For descriptive purposes, “focal” was used when immunolabeling was restricted to a limited area of the tumor, “multifocal” when immunolabeling was present in multiple spatially separated areas, and “diffuse” when immunolabeling was distributed extensively throughout the tumor. The term “heterogeneous” was used to describe areas showing variation in staining intensity. These terms were used qualitatively and were not included in the semiquantitative scoring system. For heterogeneous tumors, the intensity score was assigned according to the predominant staining intensity observed throughout the tumor section, while the proportion score reflected the overall proportion of positive neoplastic cells. The semiquantitative score was based on cytoplasmic immunoreactivity; nuclear immunoreactivity was recorded separately as a descriptive finding and was not included in the scoring.

Ki-67 expression was evaluated using digital image analysis. Stained sections were digitally scanned to enable whole-slide visualization and analysis. A single pathologist evaluated the slides using QuPath software while blinded to the histological grade and 14-3-3σ immunohistochemical scores. Areas with the highest Ki-67 immunolabeling were selected as hotspots, and tumor necrosis and non-neoplastic areas were excluded from analysis. Ki-67-positive nuclei were automatically detected by the software and manually corrected when necessary. Analysis was performed at 40× magnification, and as many fields as necessary were evaluated to obtain a minimum of 1000 neoplastic nuclei per case. The Ki-67 labeling index (LI) was calculated as the percentage of Ki-67-positive nuclei among the total number of neoplastic nuclei assessed [6,20].

### 2.4. Statistical Analysis

Statistical analysis was performed using IBM SPSS Statistics version 32.0.0 (IBM Corp., Armonk, NY, USA). Given the ordinal nature of histological grade and the small sample size, associations between histological grade, 14-3-3σ immunohistochemical score, Ki-67 labeling index, mitotic count, and estimated tumor volume were assessed using Spearman’s rank correlation coefficient (ρ) [21,22]. Corresponding 95% confidence intervals (CIs) were estimated using bias-corrected and accelerated (BCa) non-parametric bootstrap resampling with 10,000 iterations. Given the limited sample size, correlation analyses were considered exploratory and interpreted cautiously. A two-sided *p*-value < 0.05 was considered statistically significant.

Tumor dimensions were retrospectively obtained from the laboratory database based on measurements recorded during macroscopic examination (trimming) of the formalin-fixed specimens. The recorded length, width, and height of each tumor were used to estimate tumor volume according to the ellipsoid volume formula: V = (π/6) × L × W × H, where L, W, and H represent the length, width, and height of the tumor, respectively [23].

## 3. Results

Of the 12 initially identified cases, two were excluded because the immunohistochemical results were considered technically non-evaluable, leaving 10 cases for the final analysis.

### 3.1. Clinical Data

The study included 10 adult dogs diagnosed with CPPC, with a median age of 10.5 years (range, 8–17 years). Females predominated, accounting for 8/10 cases (80%), while males accounted for 2/10 cases (20%). Mixed-breed dogs were the most frequently represented (4/10; 40%), followed by Bichons (3/10; 30%), with one case each of Bernese Mountain Dog, West Highland White Terrier, and Pug. Estimated tumor volumes showed marked variability, ranging from 2.70 to 518.36 cm^3^ (median, 32.21 cm^3^). Signalment and estimated tumor volumes for individual cases are presented in Table A1.

### 3.2. Histological Examination

Histologically, of the 10 cases examined, nine (90%) were classified as papillary carcinoma (Figure S1A) and one (10%) as adenosquamous carcinoma (Figure S1B). Histological grading identified two cases (20%) as Grade I, seven cases (70%) as Grade II, and one case (10%) as Grade III. The total histological scores ranged from 7.0 to 17.0, while the mitotic count ranged from 9 to 32 mitoses per 2.37 mm^2^. Vascular invasion was identified in all cases (Figure S1C,D). The individual histological grading parameters, total scores, and corresponding histological grades are presented in Table A2.

### 3.3. Immunohistochemical Examination

**Figure 1:**
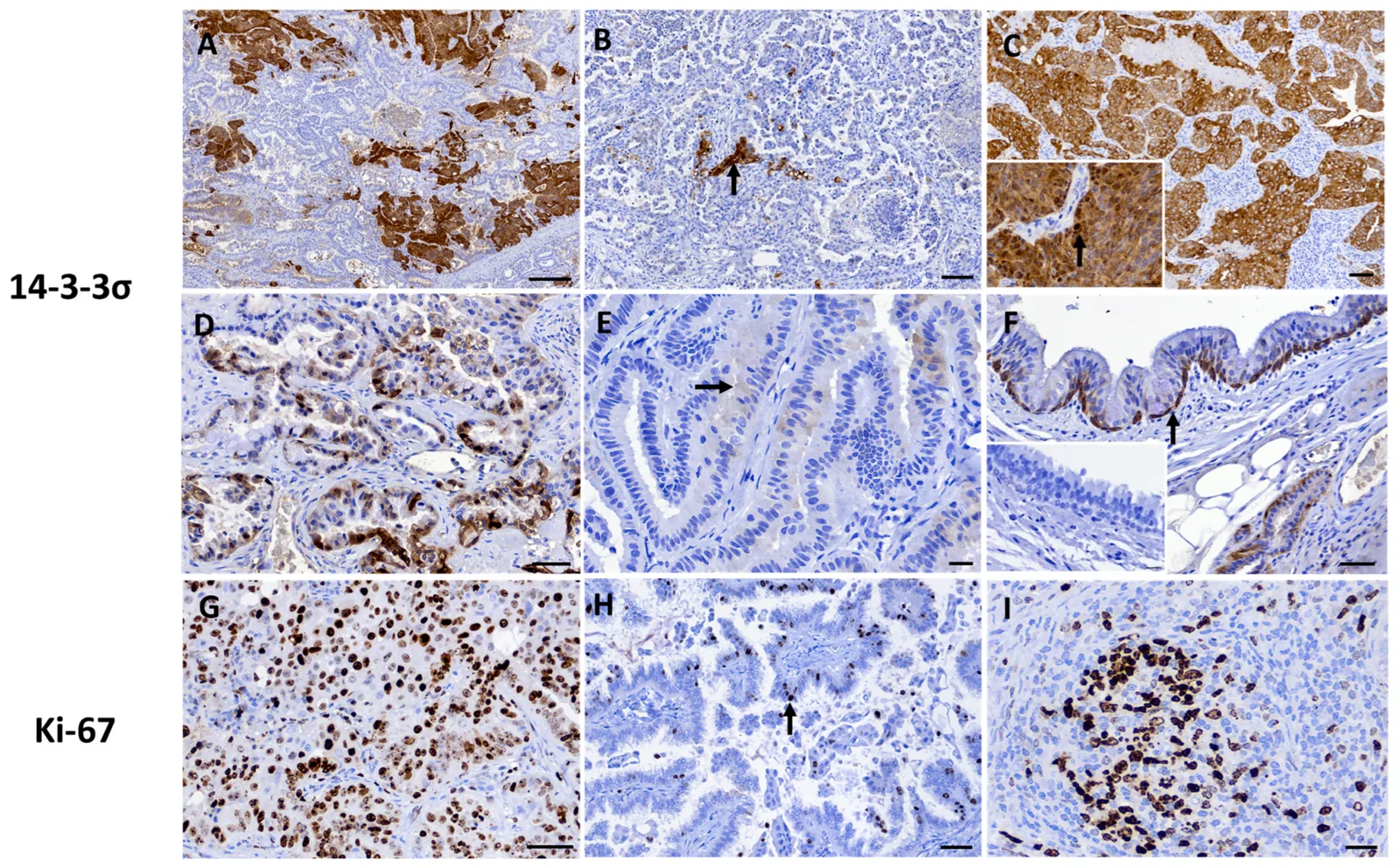
Immunohistochemical expression of 14-3-3σ and Ki-67 in CPPCs. (**A**) Multifocal and strong cytoplasmic immunolabeling for 14-3-3σ, scale bar = 250 μm; (**B**) Strong cytoplasmic immunolabeling for 14-3-3σ restricted to few neoplastic cells (arrow), scale bar = 100 μm; (**C**) Diffuse and strong cytoplasmic immunolabeling for 14-3-3σ, scale bar = 50 μm; Inset: Nuclear immunoreactivity (arrow) is also visible within areas of strong cytoplasmic staining. (**D**) Heterogeneous cytoplasmic immunolabeling with variable staining intensity for 14-3-3σ, scale bar = 50 μm; and (**E**) Focal and weak (arrow) cytoplasmic immunolabeling 14-3-3σ, scale bar = 20 μm; (**F**) Internal positive control for 14-3-3σ showing strong cytoplasmic immunolabeling in basal bronchial epithelial cells (arrow), scale bar = 50 μm; Inset: Internal negative control. (**G**) High proliferative Ki-67 labeling index in an adenosquamous carcinoma, scale bar = 50 μm; (**H**) Low proliferative Ki-67 labeling index, scale bar = 50 μm; and (**I**) Internal positive control showing Ki-67 immunolabeling in BALT, scale bar = 20 μm.

All tumors showed positive immunoreactivity for both MCK and TTF-1, supporting their epithelial phenotype and pulmonary differentiation, respectively. 14-3-3σ immunoreactivity was detected in 9/10 cases (90%), whereas 1/10 cases (10%) were negative. Multifocal staining was the predominant distribution pattern, observed in 7/9 positive cases (77.8%), whereas diffuse immunoreactivity was identified in 2/9 cases (22.2%) (Figure 1A–C). The staining pattern was predominantly heterogeneous, with variable immunoreactivity observed within individual tumor areas (Figure 1D). Among the positive cases, staining intensity was weak in 1/9 cases (11.1%), moderate in 4/9 cases (44.4%), and strong in 4/9 cases (44.4%). Despite the assigned staining intensity score, individual tumors frequently exhibited areas of weak, moderate, and strong immunolabeling (Figure 1A–E). Immunoreactivity was predominantly cytoplasmic, with nuclear staining also observed, particularly in areas showing strong cytoplasmic immunoreactivity. The overall immunohistochemical score ranged from 0 to 12. One case (10%) showed no 14-3-3σ-positive neoplastic cells and therefore had an intensity score of 0 and an overall immunohistochemical score of 0, while two cases (20%) showed the maximum score of 12. The remaining cases showed intermediate overall IHC scores of 2, 4, or 6. The internal positive control showed strong cytoplasmic 14-3-3σ immunolabeling in basal bronchial epithelial cells (Figure 1F).

The Ki-67 LI ranged from 20.4% to 89.5%, with a median value of 35.6%. Most cases showed a Ki-67 LI between 27% and 38%, whereas one case demonstrated markedly higher proliferative activity, with 89.50% of neoplastic nuclei showing nuclear immunoreactivity (Figure 1G,H). The internal positive control demonstrated Ki-67 immunolabeling in BALT (Figure 1I).

### 3.4. Statistical Analysis

**Figure 2:**
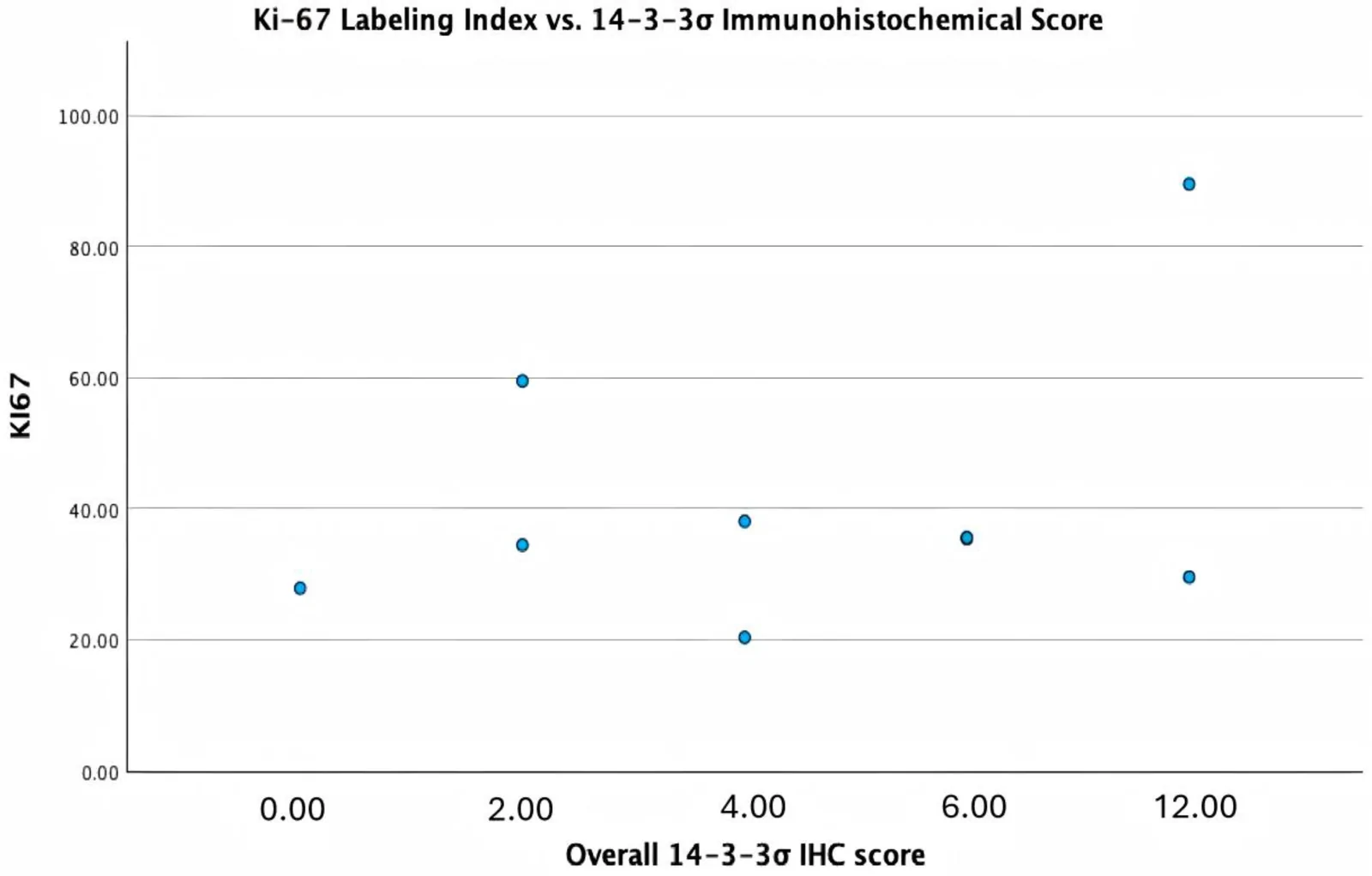
Association between 14-3-3σ immunohistochemical score and Ki-67 labeling index in primary pulmonary carcinomas. Each point represents an individual tumor. The case with the highest Ki-67 labeling index (89.5%) is shown among the individual observations.

Exploratory Spearman correlation analyses showed no statistically significant associations between 14-3-3σ immunohistochemical scores and estimated tumor volume (ρ = −0.334, 95% CI: −0.856 to 0.565, *p* = 0.345), histological grade (ρ = 0.348, 95% CI: −0.448 to 0.800, *p* = 0.324), Ki-67 index (Figure 2) (ρ = 0.273, 95% CI: −0.561 to 0.850, *p* = 0.446), or mitotic count (ρ = 0.382, 95% CI: −0.336 to 0.805, *p* = 0.275). The wide confidence intervals indicate substantial uncertainty in these estimates, and the findings should therefore be interpreted as exploratory. The association between histological grade and mitotic count was also not statistically significant (ρ = 0.622, 95% CI: 0.186 to 0.919, *p* = 0.055).

## 4. Discussion

In the present study, 14-3-3σ immunoreactivity was detected in 9 of 10 CPPCs, representing a 90% positivity rate. To the best of our knowledge, this is the first investigation to document and characterize 14-3-3σ expression in CPPCs. The high prevalence of immunoreactivity observed in these 10 cases provides preliminary descriptive data on 14-3-3σ expression in CPPCs. These findings provide an initial basis for comparison with observations reported in human pulmonary carcinomas, although differences in tumor biology, case composition, and study design should be considered [4,5,12,13,24]. In human non-small-cell lung carcinomas (NSCLC), altered 14-3-3σ expression is intimately associated with tumor progression, and its expression is frequently lost due to promoter hypermethylation or other inactivation mechanisms [12,13,24]. In contrast, we observed a 90% positivity rate in the present study group, indicating that protein expression is largely maintained. Because methylation status was not evaluated in the present study, it cannot be determined whether SFN promoter hypermethylation is absent or biologically relevant in CPPCs; the specific epigenetic mechanisms regulating 14-3-3σ in canine pulmonary tumors remain to be elucidated. Alternatively, given the ‘double-edged sword’ nature of 14-3-3σ in various human malignancies, where it can act as both a tumor suppressor and a promoter of tumor progression [10], its robust expression in these canine tumors may reflect a context-dependent biological role that diverges from the typical human NSCLC paradigm. Further molecular studies are required to determine the epigenetic regulation of the *SFN* gene in CPPCs.

The immunolabeling observed in these CPPCs was predominantly multifocal, heterogeneous, and cytoplasmic. This is consistent with the known predominantly cytoplasmic distribution of 14-3-3σ and its established involvement in cell-cycle regulation [11]. However, immunohistochemical expression alone does not demonstrate activation of a specific cell-cycle checkpoint or pathway. Following DNA damage, 14-3-3σ has been reported to contribute to G2/M cell-cycle arrest through interactions with cell-cycle regulatory proteins, including cyclin B1–CDC2 complexes [11]. More broadly, pulmonary epithelial biology involves interconnected but distinct processes including proliferation, apoptosis, and epithelial plasticity, such as epithelial–mesenchymal transition, and these processes cannot be inferred from 14-3-3σ or Ki-67 immunoreactivity alone [25,26]. The nuclear immunoreactivity observed in some areas was recorded as an additional staining pattern, but its biological significance was not investigated in the present study. Notably, similar heterogeneous expression patterns have been documented in other canine epithelial neoplasms. Previous veterinary investigations have described altered 14-3-3σ expression in canine mammary tumors, renal cell carcinomas, and gastric carcinomas [14,15,16]. In canine renal cell carcinomas, for instance, a recent study identified aberrant 14-3-3σ expression in a subset of tumors and proposed it as a potential negative prognostic factor [15]. The heterogeneous staining observed in our CPPC cases may further reflect the variable expression of 14-3-3σ across different veterinary epithelial neoplasms, although its biological significance in CPPC remains to be determined.

During the exploratory correlation analysis, positive Spearman correlation coefficients were noted between the overall 14-3-3σ immunohistochemical score and histological grade (ρ = 0.348), Ki-67 labeling index (ρ = 0.273), and mitotic count (ρ = 0.382); however, these associations lacked statistical significance. Similarly, the negative correlation regarding estimated tumor volume was not statistically significant (ρ = −0.334). Given the small sample size and the lack of statistical significance, these correlation coefficients should be interpreted strictly as exploratory observations rather than definitive evidence of a prognostic or biological relationship. The Ki-67 labeling index across our evaluated tumors ranged from 20.4% to 89.5%. Notably, the single adenosquamous carcinoma within the present case series displayed the highest Ki-67 labeling index at 89.5%. This observation is highly consistent with previous studies on spontaneous canine lung cancer, which demonstrated that adenosquamous carcinomas possess significantly higher proliferation rates than other histological subtypes [6]. Furthermore, the median proliferation index (35.6%) recorded in the present study is relatively high. This elevated value is likely a reflection of our specific methodological approach, as Ki-67 was evaluated digitally by targeting ‘hot spots’ with the highest immunolabeling, in contrast to earlier historical studies that averaged counts across random tumor fields [6]. This high baseline proliferative activity might also stem from case selection bias; because all analyzed tissues were derived from surgical pulmonary lobectomies, the study naturally selected for advanced, macroscopic lesions that required surgical intervention. Currently, there is no validated prognostic cut-off value for the Ki-67 labeling index in CPPCs, and establishing a statistically robust threshold was beyond the scope of this preliminary study due to the restricted number of cases. In human malignancies, 14-3-3σ expression frequently assumes a dual, context-dependent biological role [10]. Instead of functioning solely as a tumor suppressor, its overexpression is frequently and paradoxically associated with tumor progression, tissue invasion, metastasis, and adverse clinical outcomes [9,10]. Although positive trends emerged between 14-3-3σ expression and proliferation markers among these canine tumors, the absence of statistical significance prevents definitive conclusions and highlights the need for thorough investigation in larger study populations. It is also noteworthy that a single tumor in our case group (10%) was completely negative for 14-3-3σ immunoreactivity. Histologically, this case did not present obvious morphological deviations from the positive cases; it was classified as a Grade II carcinoma with a Ki-67 labeling index (27.9%) comparable to the group median. However, this case presented with large, estimated tumor volume (518.36 cm^3^), which was substantially larger than the other evaluated tumors. Whether this complete lack of expression represents a distinct molecular subtype characterized by the loss of 14-3-3σ [10,24], or whether it reflects advanced tumor evolution and extreme intratumoral heterogeneity within a massive lesion cannot be determined from a single case. The potential biological significance of negative 14-3-3σ expression, and its possible relationship to distinct molecular subtypes, remains an important area for future investigation in larger groups.

The retrospective nature of the study resulted in limitations in the availability and standardization of clinical information. Clinical data were obtained from available diagnostic laboratory records, and clinical signs, treatment history, and survival data were not consistently available. In addition, estimated tumor volume was derived from gross measurements of excised specimens after fixation rather than from in vivo imaging and may therefore have been affected by fixation-associated tissue shrinkage. Accordingly, correlations involving tumor volume should be interpreted cautiously, and the clinicopathological analyses should be considered exploratory rather than indicative of clinical or prognostic associations.

Because this study was based on retrospective diagnostic laboratory records, complete clinical staging and longitudinal imaging information were not available for all cases. Therefore, metastatic disease could not be excluded with absolute certainty in every case. The classification of tumors as primary pulmonary carcinomas was based on the available clinical history, histopathological features, and TTF-1 immunoreactivity.

The small sample size and uneven distribution of histological grades, with most tumors classified as Grade II and only one as Grade III, further limit the precision and interpretability of the statistical analyses. In addition, the semiquantitative scoring of 14-3-3σ may not fully capture the extent of intratumoral heterogeneity observed in some tumors. Nevertheless, this study provides preliminary descriptive data on 14-3-3σ immunoreactivity in canine primary pulmonary carcinomas. Future studies involving larger cohorts, quantitative digital image analysis [27], comprehensive clinical staging, and longitudinal clinical follow-up will be important to determine the clinicopathological and potential prognostic significance of 14-3-3σ expression in canine pulmonary carcinomas [27].

## 5. Conclusions

This preliminary study provides descriptive data on 14-3-3σ immunohistochemical expression in CPPCs. 14-3-3σ immunoreactivity was detected in 9 of 10 tumors and was predominantly multifocal, heterogeneous, and cytoplasmic, with nuclear immunoreactivity also observed. No statistically significant associations were identified between the overall 14-3-3σ immunohistochemical score and histological grade, Ki-67 labeling index, mitotic count, or estimated tumor volume. The small sample size, absence of clinical follow-up, and lack of functional validation limit the conclusions that can be drawn regarding the prognostic or mechanistic relevance of these findings. Further studies including larger numbers of cases, quantitative assessment of immunoreactivity, functional investigation, and clinical follow-up are warranted to determine whether 14-3-3σ expression is associated with specific clinicopathological features or has potential prognostic relevance in CPPC.

## Figures and Tables

**Table 1:** Antibodies used for immunohistochemical analysis.

Marker	Manufacturer	Clone	Dilution	Antigen Retrieval
Multicytokeratin (MCK)	Leica Biosystems, Melbourne, Australia	AE1/AE3	RTU	Enzymatic
Thyroid Transcription Factor-1 (TTF-1)	Leica Biosystems, Melbourne, Australia	SPT24	RTU	Citrate buffer (pH 6.0)
14-3-3σ	Santa Cruz Biotechnology, Santa Cruz, CA, USA	5D7	1:40	Citrate buffer (pH 6.0)
Ki-67	DakoAgilent Technologies, Santa Clara, CA, USA	MIB-1	RTU	Citrate buffer (pH 6.0)

## Data Availability

The original contributions presented in this study are included in the article/Supplementary Material. Further inquiries can be directed at the corresponding author.

## Supplementary Materials

The following supporting information can be downloaded at: https://www.mdpi.com/article/10.3390/vetsci13090926/s1, Figure S1: Histological features of canine primary pulmonary carcinomas (H&E).

## Abbreviations

The following abbreviations are used in this manuscript:

BALT	Bronchus-associated lymphoid tissue
BCa	Bias-corrected and accelerated
CI	Confidence interval
COX-2	Cyclooxygenase-2
CPPC	Canine primary pulmonary carcinoma
DAB	3,3′-diaminobenzidine
EGFR	Epidermal growth factor receptor
FFPE	Formalin-fixed, paraffin-embedded
H&E	Hematoxylin and eosin
HPF	High-power field
IHC	Immunohistochemistry/Immunohistochemical
LI	Labeling index
MCK	Multicytokeratin
NSCLC	Non-small-cell lung carcinoma
PCNA	Proliferating cell nuclear antigen
RTU	Ready to use
SFN	Stratifin
TTF-1	Thyroid transcription factor-1

## Appendix A

**Table A1:** Signalment, tumor dimensions, Ki-67 and 14-3-3σ expression in CPPC.

Case ID	Breed	Age (Years)	Sex	Estimated Tumor Volume (cm^3^)	Ki-67 Labeling Index (%)	14-3-3σ Proportion Score	14-3-3σ Staining Intensity Score	14-3-3σ Staining Distribution	Overall 14-3-3σ IHC Score
1	Bernese Mountain Dog	10	F	518.36	27.9	1	0	Not applicable	0
2	West Highland White Terrier	13	F	32.99	59.5	2	1	Multifocal	2
3	Mixed breed	11	M	113.1	38.1	2	2	Multifocal	4
4	Bichon	10	M	28.27	35.5	2	3	Multifocal	6
5	Mixed breed	17	F	2.7	35.5	3	2	Multifocal	6
6	Mixed breed	12	F	31.42	34.5	1	2	Multifocal	2
7	Bichon	13	F	125.66	29.6	4	3	Diffuse	12
8	Bichon	9	F	6.7	89.5	4	3	Diffuse	12
9	Pug	10	F	44.64	35.6	2	3	Multifocal	6
10	Mixed breed	8	F	15.71	20.4	2	2	Multifocal	4

Note: 14−3−3σ proportion score: 1 = ≤10% positive cells, 2 = 11–50%, 3 = 51–80%, and 4 = >80%. 14−3−3σ staining intensity score: 0 = negative, 1 = weak, 2 = moderate, and 3 = strong. The overall 14−3−3σ immunohistochemical score was calculated by multiplying the proportion and intensity scores. Ki−67 labeling index was calculated as the percentage of Ki−67-positive nuclei among the total number of nuclei assessed and is presented to one decimal place. Abbreviations: M- Male; F- Female.

**Table A2:** Histological grading parameters and scores of CPPC.

Case ID	Overall Differentiation	Nuclear Pleomorphism	Mitotic Count (2.37 mm^2^)	Mitotic Count Score	Nucleolar Size	Tumor Necrosis	Tumor Fibrosis	Demarcation	Total Score	Histological Grade
**1**	2	1	11	2	0.5	1	0.5	2	9.0	2
**2**	2	1	9	1	0.5	1	0.5	1	7.0	1
**3**	2	2	12	2	1.0	2	0.5	1	10.5	2
**4**	2	2	32	4	1.0	2	1.0	2	14.0	2
**5**	1	1	11	2	1.0	1	0.0	1	7.0	1
**6**	1	1	18	2	0.5	2	0.5	1	8.0	2
**7**	3	3	22	3	1.5	1	1.0	3	15.5	3
**8**	2	3	11	2	1.5	1	0.5	1	11.0	2
**9**	2	2	16	2	0.5	1	1.0	3	11.5	2
**10**	3	3	10	1	1.5	1	1.0	3	13.5	2

Note: The criteria for this histological grading system were adapted from Avallone et al. [20]. The resulting grades served strictly a descriptive purpose and were not considered validated as prognostic markers for CPPC. Mitotic count scores were assigned based on the number of mitoses per 2.37 mm^2^ as follows: score 1 (1–10 mitoses); score 2 (11–20 mitoses); score 3 (21–30 mitoses); and score 4 (≥31 mitoses). The remaining histological parameters were scored as follows: Overall differentiation: 1 = Well-differentiated (orderly arrangement of neoplastic cells); 2 = Moderately differentiated (mixed areas of orderly and lost organization); 3 = Poorly differentiated (loss of orientation and polarity). Nuclear pleomorphism: 1 = Mild (uniform nuclei); 2 = Moderate (<2-fold difference in size); 3 = Severe (>2-fold difference and irregular shapes). Nucleolar size: 0.5 = Small; 1.0 = Medium (identifiable but not prominent); 1.5 = Large (prominent, ≥1/3 nucleus size). Tumor necrosis: 0 = None; 1 = 1% to 20%; 2 = 21% to 50%; 3 = >50%. Tumor fibrosis: 0 = None; 0.5 = 1% to 20%; 1.0 = 21% to 50%; 1.5 = >50%. Demarcation: 1 = Well-demarcated (sharp border, no capsule); 2 = Moderately demarcated (protrusions into adjacent tissue); 3 = Invasive (borders not distinguishable).
