# Supplementary material for: Immunohistochemical Characterization of 14-3-3σ Expression in Canine Primary Pulmonary Carcinomas: A Preliminary Study
Source: Vet Sci. 2026 Sep 9;13(9):926. doi: 10.3390/vetsci13090926 (PMC13611917; doi:10.3390/vetsci13090926)
Supplement: Supplementary file 1 [file vetsci-13-00926-s001.zip › vetsci-4537000-supplementary.pdf]

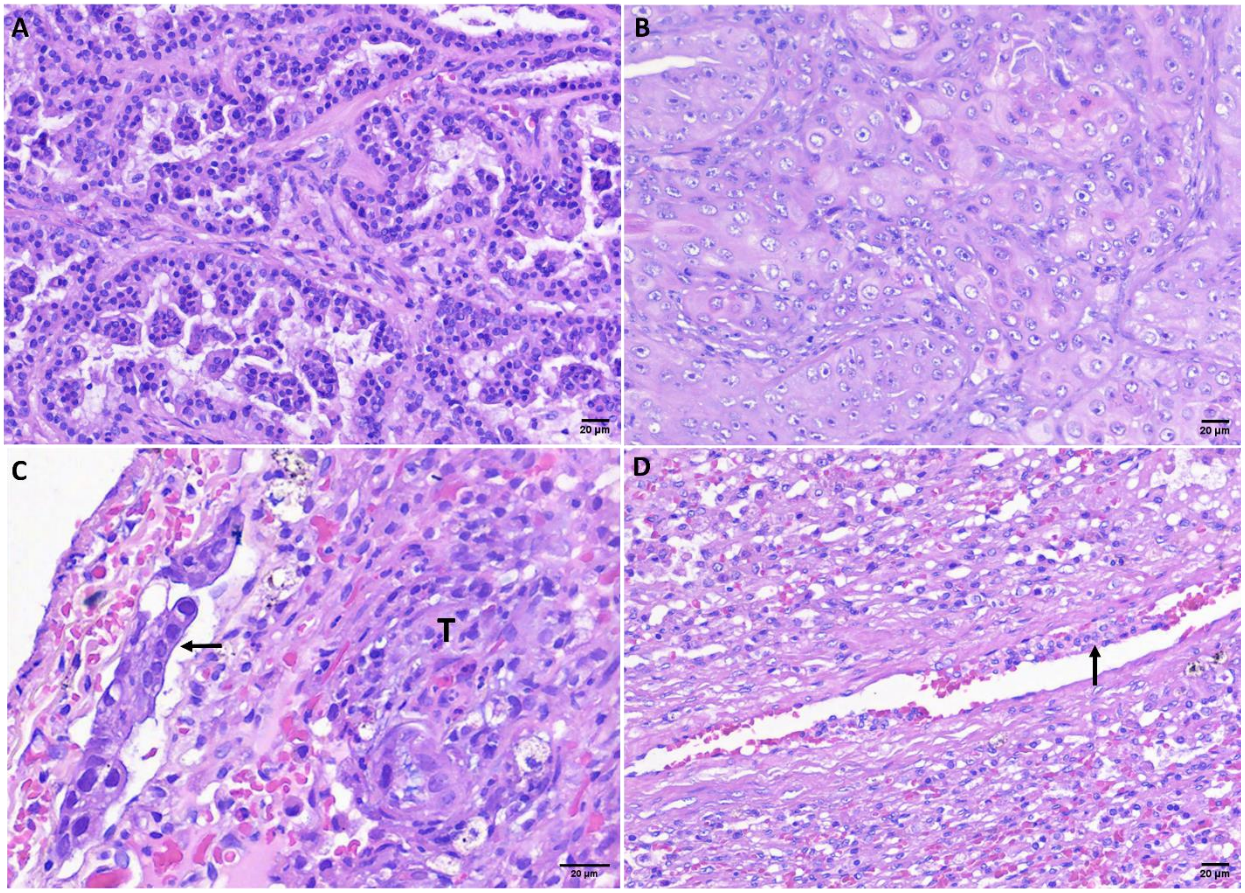

Figure S1. Histological features of canine primary pulmonary carcinomas (H&E). (A) Adenocarcinoma exhibiting a papillary pattern, scale bar= 20 µm. **(B)** Adenosquamous carcinoma, scale bar= 20 µm. **(C)** Thin-walled vascular structure, likely representing a lymphatic vessel, containing a neoplastic embolus (arrow) adjacent to the primary tumor (T), scale bar= 20 µm. **(D)** Blood vessel containing a neoplastic embolus (arrow), scale bar= 20 µm.
